# Where is the difference between an epidemic and a high endemic level with respect to nosocomial infection control measures? An analysis based on the example of vancomycin-resistant Enterococcus faecium in hematology and oncology departments

**DOI:** 10.3205/dgkh000299

**Published:** 2017-08-28

**Authors:** Nikos Ulrich, Petra Gastmeier

**Affiliations:** 1 Institute for Hygiene and Environmental Medicine, Charité – University Medicine, Berlin, Germany

**Keywords:** outbreaks, endemic, vancomycin-resistant enterococi, haematology, oncology

## Abstract

Some infection control recommendations distinguish epidemic and endemic levels for infection control. However, it is often difficult to separate long lasting outbreaks from high endemic levels and it remains open, if this distinction is really useful.

**Aim:** To compare infection control measures in endemic and epidemic outbreaks.

**Methods:** The example of vancomycin-resistant *Enterococcus faecium* outbreaks in haematology or oncology departments was used to analyse differences in infection control measures between outbreaks and high endemic levels. The outbreak database and PubMed, including long lasting outbreaks, were used for this analysis. Two time limits were used for separation: 6 and 12 months. In addition, monoclonal and polyclonal outbreaks were distinguished.

**Findings:** A total of 36 outbreaks were included. 13 outbreaks lasted 6 months or less, 9 outbreaks more than 6 months but at maximum 12 months and 9 more than 12 months. For the remaining outbreaks, no information about their duration was available. Altogether, 11 outbreaks were monoclonal and 20 polyclonal. Considering infection control measures, there were almost no differences between the different groups compared. Patient screening was given up in 37.5% of long lasting outbreaks (>12 months) and hand hygiene not reported in the majority of polyclonal outbreaks (77.8%).

**Conclusion:** Despite many institutions trying to add further infection control measures in case of an outbreak, evidence based infection control measures should be implemented in endemic and epidemic situations. The crucial aspect is probably the degree of implementation and its control in both situations.

## Zusammenfassung

Einige Krankenhaus-Hygieneempfehlungen unterscheiden zwischen epidemischen und endemischen Niveaus bei Infektionskontrollmaßnahmen. Oft ist es schwer, zwischen lang andauernden Ausbrüchen und einem hohen endemischen Niveau zu unterscheiden und es bleibt unklar, ob diese Unterscheidung sinnvoll ist.

**Ziel:** Vergleich von Infektionskontrollmaßnahmen bei endemischen und epidemischen Ausbrüchen.

**Methoden:** Das Beispiel von Vancomycin-resistentem *Enterococcus faecium* auf hämatologischen und onkologischen Stationen wurde verwendet, um die unterschiedlichen Infektionskontrollmaßnahmen bei Ausbrüchen und bei hohen endemischen Niveaus zu vergleichen. Für die Analyse wurden die Outbreak-Datenbank sowie PubMed verwendet. Es wurden zwei zeitliche Grenzen zur Unterscheidung von epidemischen und endemischen Situationen gesetzt: 6 und 12 Monate. Zusätzlich wurden monoklonale und polyklonale Ausbrüche unterschieden.

**Ergebnis:** Insgesamt wurden 36 Artikel in die Analyse aufgenommen. 13 Ausbrüche hatten eine maximale Dauer von 6 Monaten, 9 Ausbrüche dauerten länger als 6 Monate, aber nicht länger als 12 Monate und 9 Ausbrüche dauerten länger als 12 Monate. In den verbliebenden Ausbrüchen wurde keine Angabe zur Dauer gemacht. Insgesamt waren 11 Ausbrüche monoklonal und 20 polyklonal. Bezüglich der Infektionskontrollmaßnahmen gab es nahezu keinen Unterschied zwischen den beiden Gruppen. Patientenscreening wurde in 37,5% der langen Ausbrüche (>12 Monate) nicht durchgeführt und über verstärkte Händehygiene wurde in der Mehrheit der polyklonalen Ausbrüche (77,8%) nicht berichtet.

**Schlussfolgerung:** Obwohl viele Institutionen versuchen, weitere Infektionskontrollmaßnahmen im Fall eines Ausbruchs hinzuzufügen, sollten Evidenz basierte Maßnahmen in epidemischen und endemischen Situationen eingesetzt werden. Offensichtlich ist der entscheidende Punkt, in welchem Maß die Infektionskontrollmaßnahmen umgesetzt werden und wie die Umsetzung kontrolliert wird.

## Introduction

Some infection control guidelines distinguish control measures in endemic and epidemic conditions [1], [2], [3]. In addition, some authors require that future studies have to differentiate between epidemic and endemic situations in order to adjust prevention strategies for the individual settings [4]. However, often it is not clear, if an outbreak is continuing or if it should be categorized a high endemic level. In the literature, one can sometimes find terms such as “sustainable endemic outbreak” or “prolonged outbreak” [5], [6], [7], [8]. It is also difficult to understand, why different infection control measures are recommended for both outbreak situations and endemic conditions. If a measure has shown to be effective in decreasing the risk of transmission or the risk of infection based on scientific literature, it should be applied. 

Difficulties in distinguishing outbreaks and high endemic levels of nosocomial pathogens occur very often for example in the case of vancomycin-resistant *Enteroccus faecium* (VRE) in haematology and oncology departments. In this patient group asymptomatic colonization of the gastrointestinal tract is more common than clinically recognized infection by a ratio of 10:1 [9]. As a consequence, situations with a large number of VRE colonizations are often not recognized as a problem and not considered a real outbreak. However, in particular bloodstream infections due to VRE are associated with substantial morbidity and mortality. Even under the conditions of modern VRE therapies, mortality is almost twice as high when the pathogen causing blood stream infection is a VRE compared with Vancomycin susceptible *E. faecium* [10], [11]. That means outbreaks in this patient group are a serious problem. Therefore we want to use this example to answer the question whether a distinction between epidemic and endemic conditions for infection control measures is really useful.

## Methods

Primarily, we used the outbreak database to investigate this question. It contains not only many outbreaks but also many sustained and prolonged outbreaks (often over 2 years) which normally should be regarded as a high endemic level. 

The Outbreak Database (http://www.outbreakdatabase.com) is a database containing nosocomial outbreaks worldwide and is currently the largest collection of nosocomial outbreaks [12]. The database contains information from nosocomial outbreaks in a standardized format. Parameters on several levels can be set in order to obtain more specific search results. In this case, parameters have been set to only include articles that contain ‘vancomycin-resistant *Enterococcus faecium*’ as the microorganism and ‘haematology/oncology’ as the location. The articles found in the outbreak database in February 2017 were then reviewed and a manual search of reference lists of these articles was conducted and, if appropriate, included in our study. To identify additional articles which are not yet filed in the outbreak database, but also relevant to the topic of interest, two additional searches of PubMed were performed on the same day using the following combination of MeSH terms:

- [hematology] AND [vancomycin-resistant *Enterococcus faecium*]
- [oncology] AND [vancomycin-resistant *Enterococcus faecium*]

When available the following items were extracted from each VRE outbreak: duration of the outbreak, if typing was performed and the infection control measures applied.

To distinguish short and long outbreaks (high endemic levels), two different definitions for the duration of a short outbreak were used: at maximum 6 months and 12 months. Due to monoclonal clusters sometimes being considered as an outbreak and polyclonal clusters as a high endemic level, we also used the typing information of the outbreaks as a distinction. Most of the outbreaks we found were not really monoclonal. Often, in addition to a dominating strain, one or two other strains were found. Therefore, we considered an outbreak as mainly monoclonal if more than 75% of strains were indistinguishable.

The statistical analyses to compare the two groups were performed with ‘open epiInfo’ using Fisher’s exact test.

## Results

Our search yielded 36 outbreaks appropriate for this study [13], [14], [15], [16], [17], [18], [19], [20], [21], [22], [23], [24], [25], [26], [27], [28], [29], [30], [31], [32], [33], [34], [35], [36], [37], [38], [39], [40], [41], [42], [43], [44], [45], [46], [47], [48], [49]. The mean duration of 31 outbreaks with information about its duration was 11 months (range 1 to 36 months). Molecular typing was performed in 31 articles mainly using pulse field gel electrophoresis (PFGE). 

On average 4.5 infection control measures per outbreak were employed (range 1 to 9). The most frequent measure adopted was patient screening in 28 outbreaks, followed by isolation/cohorting in 21 outbreaks. 14 outbreaks involved environmental screening and in 15 outbreaks intensified cleaning and disinfection of the environment was reported. Six outbreaks reported the closure of the affected location. A full overview of the extracted data is given in Table 1 (Tab. 1). 

Table 2 (Tab. 2) provides the infection control measures according to the duration of the outbreaks. Whereas patient screening is performed in all short outbreaks, it was not always performed in long lasting outbreaks (37.5%). This difference is significant when the limit of up to a maximum of 12 months was used. For all other infection control measures, no difference between both groups was found. 

Table 3 (Tab. 3) shows the distribution according to mainly monoclonal and polyclonal outbreaks. There is no association between clonality and outbreak duration and also almost no influence on infection control measures. There is only one exception: Hand hygiene played a greater role in monoclonal outbreaks and was not reported in 77.8% of polyclonal outbreaks.

## Discussion

Normally, during an outbreak, all relevant infection control measures should be applied to end the outbreak as soon as possible. However, if the implementation of infection control measures is insufficient, the same measures have to be used over a long period. In the case of a monoclonal outbreak, one might argue that the implementation of infection control measures is better in order to eliminate this specific strain as quickly as possible. On the other hand, polyclonal outbreaks provide evidence that not a specific strain with a high potential for transmission is available, but rather that a general infection control problem may exist on this ward or department.

The implementation of infection control measures also depends on the scientific evidence for these measures. In general, there is only little evidence for effective infection control measures to decrease VRE transmission and the quality of the available studies is rather low. In a meta-analysis, only hand hygiene was associated with a 47% decrease in the VRE acquisition rate while contact precautions did not significantly reduce the VRE acquisition rate [48]. Therefore, the infection control measures found in the included outbreaks represent the infection control measures normally recommended in situations with a high number of patients with multiresistant organisms and in an immunocompromised patient group [1], [9], [49], [50].

Patient screening was the most common infection control measure. It is important to detect patients with VRE early on to be able to prevent it spreading among patients. Our data for long lasting outbreaks (>12 months) show, that patient screening was given up on or not introduced at all in 37.5% of these outbreaks. The reason may be the lacking possibility to isolate and cohort all identified patients. Interestingly, hand hygiene was not reported in the majority of polyclonal outbreaks, despite being the single most important measure to stop transmission. In general hand hygiene has been emphasized in 11 articles of our review only, which is fewer than expected. This might be due to a general underreporting of enforced hand hygiene as an infection control measure, despite its use during an outbreak. Another measure that was surprisingly seldom mentioned was antibiotic stewardship. At least in longer lasting outbreaks and in polyclonal outbreaks this seems to be one of the most important interventions, but was only reported in 37.5% and 27.8% of cases respectively [51], [52], [53].

The review has a number of limitations. First, perhaps the example VRE may not be representative for other outbreaks, but it is very often associated with longer duration and was therefore selected. 

Second, the two definitions to distinguish short and long outbreaks and the definition of mainly monoclonal outbreaks were mainly chosen to create groups with a similar number of outbreaks in both groups to be used for comparison. However, the tables show that the infection control measures are almost the same in the various groups. 

Third, the majority of articles used PFGE or PCR as the microbiological tool to assess strain relatedness. Many of the outbreaks are from the 1990s, where whole genome sequencing (WGS) was not yet available. While PFGE is a reliable method for the detection of strain relatedness during nosocomial outbreaks, WGS offers an even more precise strain differentiation and is meanwhile often used in VRE outbreak investigations [54], [55].

Fourth, the number of VRE outbreaks considered in our review may be too small to identify further relevant differences between the different groups investigated.

Finally, there is still no uniform reporting of outbreaks as required by the ORION statement [56]. Therefore, it may be the case, that some infection control measures were used but not mentioned. 

In addition and probably most important, the degree of implementation of infection control measures is a key aspect and it is impossible to derive from the outbreak description how rigorously the measures were implemented and if implementation was controlled. 

In conclusion, according to our example with a relatively large number of short and long lasting outbreaks, it was impossible to identify relevant differences among infection control measures between short outbreaks and high endemic levels as well as between monoclonal and polyclonal outbreaks. Therefore, we believe the distinction of the two groups in infection control guidelines does not reflect the current situation in hospitals and may not be very helpful.

## Notes

### Competing interests

On behalf of all authors, the corresponding author states that there is no conflict of interest.

### Funding source

This research did not receive any specific grant from funding agencies in the public, commercial, or not-for-profit sectors.

## Figures and Tables

**Table 1:**
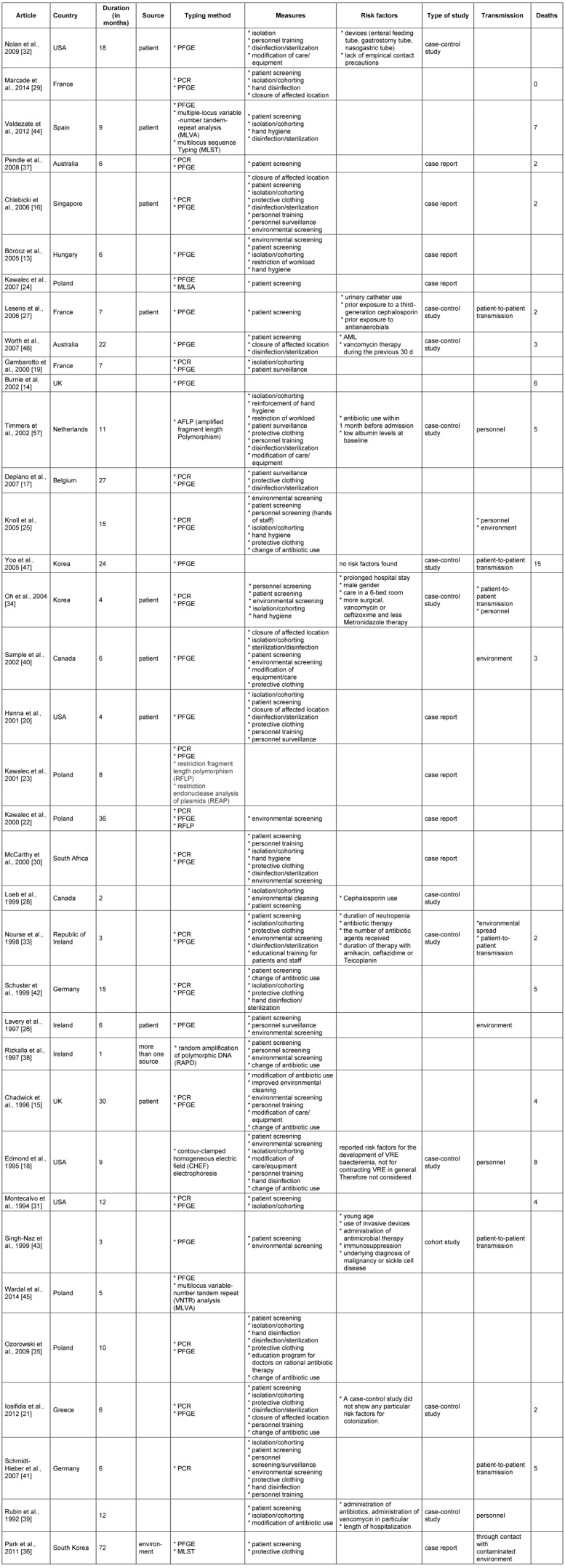
Overview of all extracted data from 36 outbreaks

**Table 2:**
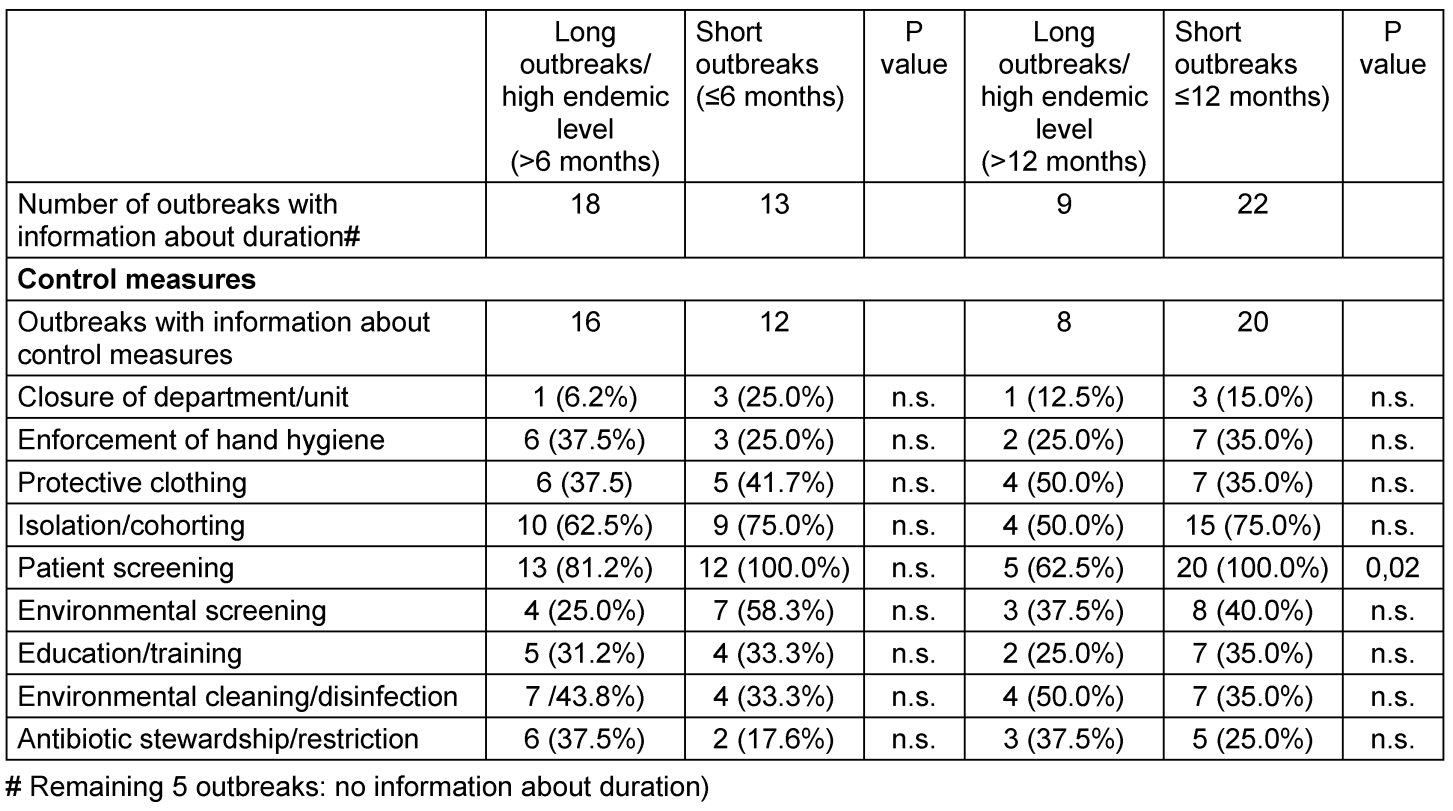
Comparison of long and short outbreaks according to two thresholds (maximum duration of a short outbreaks ≤6 months and ≤12 months)

**Table 3:**
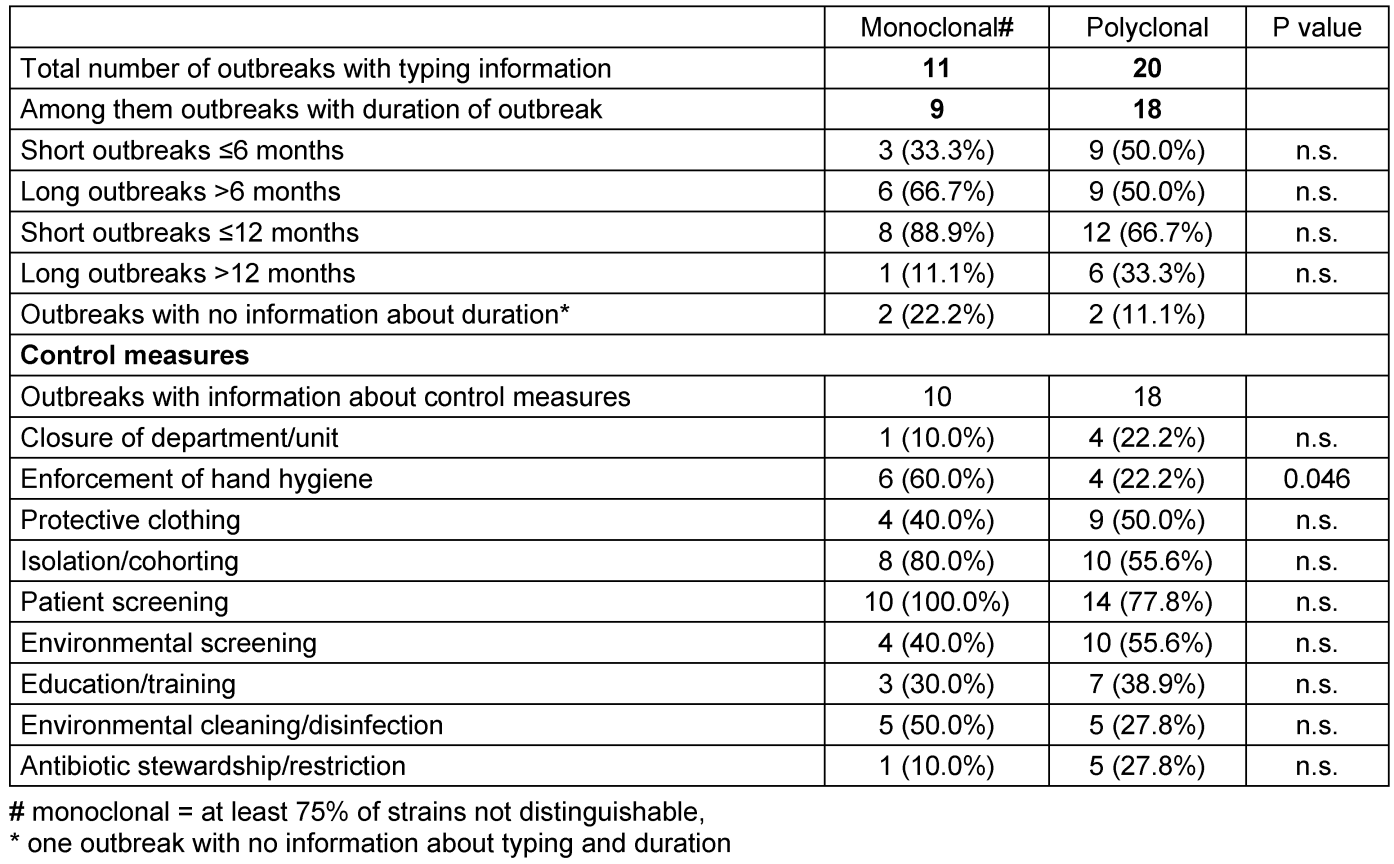
Comparison of polyclonal and monoclonal outbreaks
